# The Transcription Factor PfAP2-O Influences Virulence Gene Transcription and Sexual Development in *Plasmodium falciparum*

**DOI:** 10.3389/fcimb.2021.669088

**Published:** 2021-06-28

**Authors:** Eliana F. G. Cubillos, Isadora Oliveira Prata, Wesley Luzetti Fotoran, Lisa Ranford-Cartwright, Gerhard Wunderlich

**Affiliations:** ^1^ Department of Parasitology, Institute for Biomedical Sciences, University of São Paulo, São Paulo, Brazil; ^2^ Institute of Biodiversity, Animal Health & Comparative Medicine, College of Medical, Veterinary and Life Science, University of Glasgow, Glasgow, United Kingdom

**Keywords:** PfAP2-O, virulence factor, var, antigenic variation, epigenetic memory

## Abstract

The human malaria parasite *Plasmodium falciparum* expresses variant PfEMP1 proteins on the infected erythrocyte, which function as ligands for endothelial receptors in capillary vessels, leading to erythrocyte sequestration and severe malaria. The factors that orchestrate the mono-allelic expression of the 45–90 PfEMP1-encoding *var* genes within each parasite genome are still not fully identified. Here, we show that the transcription factor PfAP2-O influences the transcription of *var* genes. The temporary knockdown of PfAP2-O leads to a complete loss of *var* transcriptional memory and a decrease in cytoadherence in CD36 adherent parasites. AP2-O-knocked-down parasites exhibited also significant reductions in transmission through *Anopheles* mosquitoes. We propose that PfAP2-O is, beside its role in transmission stages, also one of the virulence gene transcriptional regulators and may therefore be exploited as an important target to disrupt severe malaria and block parasite transmission.

## Introduction

The human malaria parasite *Plasmodium falciparum* remains a risk to a significant portion of the world’s population. Despite the considerable success of control measures, *P. falciparum* still causes around 400,000, deaths per year, mainly in the sub-Saharan African countries, where most of the victims are either pregnant women or children under 5 years of age (World Health Organization, 2019). Common approaches for malaria control and elimination are artemisinin-based combination therapies (ACT) to treat blood stages in infected patients and insecticide-impregnated bed nets to prevent mosquito bites and parasite transmission. Although a significant reduction of malaria cases during the first decades of implementation of the aforementioned measures was achieved, recent studies have demonstrated the emergence of parasites resistant to almost all antimalarial treatments including artemisinin (Dondorp et al., 2009) and mosquitoes’ resistance to the most commonly-used insecticides—pyrethroids, organochlorines, carbamates, and organophosphates—across different endemic areas (WHO, 2020). Therefore, the study and characterization of new drug targets to interfere in parasite transmission are crucial to achieve long-term malaria eradication (Delves et al., 2018).

A major parasite virulence factor is PfEMP1 (*Plasmodium falciparum* erythrocyte membrane protein 1), which consists of proteins expressed on the infected red blood cell surface, encoded by the multicopy *var* gene family (Su et al., 1995). Based on features such as chromosomal location, protein domain structure, and sequence similarities, *var* genes have been classified into three major groups, termed (upsA, upsB, and upsC), two intermediate groups, (upsB/A and upsB/C), and two distantly related genes *var1csa* and *var2csa* (upsD and upsE respectively) (Lavstsen et al., 2003; Joergensen et al., 2010). The *var* genes are expressed in a timely, controlled manner so that only one or a few *var* genes are transcribed per parasite while all others are transcriptionally silenced, which means that only one PfEMP1 type is exported to the erythrocyte surface (Chen et al., 1998; Scherf et al., 1998). Eventually, by a mechanism known as switching, the active *var* gene is silenced and another one starts to be transcribed so that a different type of PfEMP1 is exported (Scherf et al., 1998). This phenomenon known as mutually allelic exclusion leads to the characterized PfEMP1 antigenic variation and is one of the most important strategies to avoid the host immune system (Chen et al., 1998).

The described mechanisms controlling changes in *var* transcription profiles, and how this switching is orchestrated, are mainly associated with chromatin modifications in *var* promoter regions. It has been shown that the active *var* gene is marked by the acetylation of lysine 9 (H3K9ac) and the presence of a special histone H2.AZ (Petter et al., 2011), while the repressed genes are marked by the trimethylation of the Histone 3 Lysine 9 (H3K9me3) (Lopez-Rubio et al., 2007). Additionally, the selected *var* allele that is transcribed in the next cycle (poised state) is epigenetically marked by demethylation of the Histone 3 lysine 4 (H3K4me3 to H3K4me2) (Lopez-Rubio et al., 2007). There are different proteins responsible for reading, marking, or removing these histone modifications. The histone deacetylases PfSIR2A and PfSIR2B were found to have a repressive role in upsA and upsB type genes, respectively (Duraisingh et al., 2005; Freitas et al., 2005; Tonkin et al., 2009). Also, the methyltransferases PfSET10 and PfSETvs are associated with *var* gene transcription. While PfSET10 seems to be responsible for the maintenance of the H3K4 methylation mark in the active *var* gene during the poised state (Volz et al., 2012), PfSETvs was found to be a repressor of *var* transcription when knockout parasites for this protein displayed virtually all *var* transcripts in the parasite nucleus (Jiang et al., 2013). The histone-mark-reading protein, Heterochromatin protein 1 (PfHP1) is associated with silenced chromatin around the promoter region of *var*, and also with genes involved in the production of sexual/transmission stages of the parasite, known as gametocytes (Pérez-Toledo et al., 2009; Brancucci et al., 2014). Accordingly, PfHP1 knockdown leads to transcriptional de-repression of *var* genes, disruption of allelic exclusion, and the induction of gametocytogenesis, indicating a link between *var* gene transcription and transmission (Brancucci et al., 2014). Regulatory elements in promoter regions of *var* genes are also involved in transcription regulation. It was shown that each *var* gene has two functional promoters, one in the exon I and the other within the intron, which seems to be necessary for repression of *var* transcription (Calderwood et al., 2003). Some long non-coding RNAs, (ncRNAs) that are transcribed from telomere repeats were proposed to influence *var* transcription, at least those in the subtelomeric region (Sierra-Miranda et al., 2012), and sense and antisense long ncRNAs transcribed from the *var* intron may have a regulatory effect (Epp et al., 2009). Furthermore, the exosome related RNAse PfRrP6 seems to fine-regulate the degradation of these RNAs (Fan et al., 2020), and the specific DNA helicase, RecQ1, was suggested to be involved in *var* transcription when its knockdown increased the *var* recombination levels and high rates of chimeric *var* genes were observed with actively transcribed *var* loci (Li et al., 2019). Finally, the chromatin remodeler, PfISWI, was suggested to play a key role in the activation of *var* genes (Bryant et al., 2020).

Members of the ApiAP2 family of plant-like transcription factor proteins have been associated with important processes throughout the entire life-cycle of *Plasmodium*. In the rodent malaria parasite *P. berghei*, parasites lacking the protein encoded by *PbAP2-Sp* (PF14_0633/PF3D7_1466400 *P. falciparum* ortholog) were unable to complete the sexual cycle inside the mosquito, as they could not form sporozoites (Yuda et al., 2010). Also in *P. berghei*, parasites lacking *PbAP2-O* (Pf11_0442/PF3D7_1143100 *P. falciparum* ortholog) developed as aberrant ookinetes that were unable to invade the *Anopheles* midgut and form oocysts (Yuda et al., 2009). In *P. falciparum*, the disruption of *PfAP2-G* (PF3D7_1222600) resulted in a complete loss of gametocyte formation, whereas increased expression led to the increased induction of gametocyte-commitment (Kafsack et al., 2014). Campbell and colleagues identified domains in three ApiAP2 members (Balaji et al., 2005) that showed affinity to specific domains in *var* 5′ upstream (5′ ups) regions (Campbell et al., 2010). One of them (PF3D7_0604100/PfSIP2) was shown to interact with upsB *var* sequences, and it was suggested that this protein has a role in tethering of chromatin (Flueck et al., 2010). For the second gene, Martins and colleagues demonstrated an upregulation of clonally-variant genes (*stevor* and *rif*, but not *var*) when the predicted protein, PfAP2-exp was truncated (Martins et al., 2017). The role of the third protein, PF3D7_1143100/Pf11_0442, (PbAP2-O ortholog) remains unknown in *P. falciparum*. Here, we addressed the function of this protein, named PfAP2-O, in the asexual and sexual cycles of *P. falciparum* and found that it influences transcription of *var* genes and is crucial for parasite transmission to *Anopheles* mosquitoes.

## Material and Methods

### *P. falciparum* Culture

*P. falciparum* NF54 parasites and transgenic strains were cultured in 4% hematocrit in RPMI supplemented with 0.25% Albumax 1 (Invitrogen)/5% human plasma under a 90% N_2_, 5% CO_2_, 5% O_2_ atmosphere. Culture medium was changed daily or every two days, and parasites were supplemented with fresh blood every 4 days or more often depending on the parasitemia in the culture. The strains containing a destabilizing domain were maintained in the presence of Shield-1 at 0.5 µM that stabilizes the target protein (de Azevedo et al., 2012).

In order to obtain synchronized cultures, mature stage parasites were frequently (every 3 weeks) purified using Voluven 6% (Fresenius Kabi) using the method published by Lelievre and colleagues (Lelièvre et al., 2005). When ring stage parasites were required, cultures with predominantly this form (up to 8 h after reinvasion) were treated for 10 min with 20 volumes (of the erythrocyte volume) 5% sorbitol solution at room temperature (Lambros and Vanderberg, 1979).

### Molecular Cloning/Plasmid Construction

**Figure 1:**
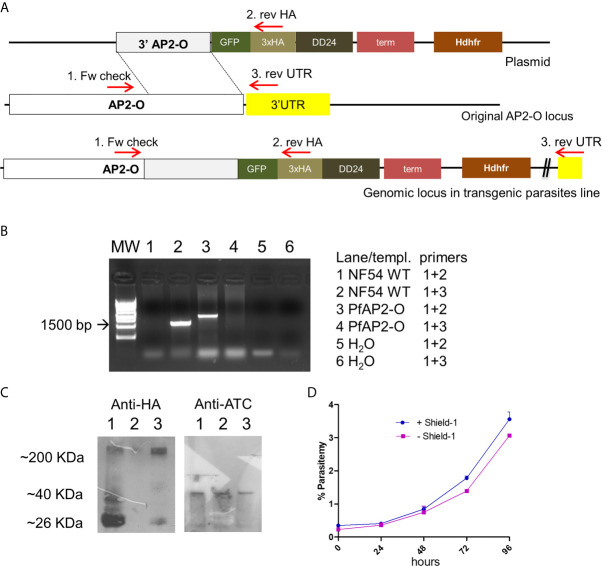
PfAP2-O modification and subsequent partial knockdown leads to no discernible growth phenotype. **(A)** The plasmid structure and its integration of tags by homologous recombination after transfection and cloning are shown. **(B)** PCR analysis of genomic DNA from the recombinant parasite line confirms the integration of the plasmid and absence of wild type versions in the target gene. Combination of Oligonucleotides 3 + 1 results in the amplification of unmodified locus and the band corresponding to oligonucleotides 1 + 2 are the results of modified AP2-O gene. Note that there is no PCR fragment visible in lane 4 which would indicate residual non-modified AP2-O loci. **(C)** Western blot using an antiHA antibody to detect PfAP2-O-GFP expression in parasites submitted to temporary knockdown by the absence of Shield-1. Lane 1: Parasites grown in the presence of Shield-1. Lane 2: Parasites grown for two reinvasions without Shield-1. Lane 3: Parasites after re-establishment of PfAP2-O-GFP by re-addition of Shield-1 for two reinvasion cycles. As a loading control, a polyclonal antiATC (plasmodial aspartate transcarbamoylase) was used (see **Supplementary Material** for the full exposure). **(D)** Growth curves from NF54::PfAP2-O_GFP_HA_DD24 with and without 0.5 µM Shield-1 (data from triplicates).

*P. falciparum* knock-in expression plasmids were constructed using the PfAP2-O 3′-end portion. This region was cloned into the transfection plasmid p_GFP_HA_DD24 (**Figure 1A**). First, genomic DNA (gDNA) was prepared from NF54 parasites using the Proteinase K/Phenol-Chloroform method described in Methods in Malaria Research (Ljungström et al., 2008). The final DNA pellet was resuspended in TE and used as a template for PCR using oligonucleotide primers PfAP2-O fw: 5′-AGATCTGAATTGTTCAGAACATTTAAATATAGTACC and PfAP2-O rev: 5′-CTGCAGTAAATTATTAAGGGGGATGTTATTATTAAC (restriction sites BglII and PstI underlined).

The oligonucleotides were used in standard PCRs (30 cycles of 94°C, 40s, 50°C for 40s and 65°C for 90s, final polymerization of 10 min at 65°C). The PCR products were excised and purified *via* the glassmilk method (Boyle and Lew, 1995), ligated into pGEM T easy (Promega) and transformed in DH10B chemically competent *E. coli* cells. After plating and growth, colonies were grown in Terrific Broth—ampicillin (TB-amp) supplemented liquid medium and the plasmids extracted using a standard miniprep protocol (Green and Sambrook, 2012). Clones were sequenced by the dideoxy-dNTP method and checked for their integrity. The correct fragment was subcloned in the p_GFP_HA_DD24 vector *via* PstI/BglII. Recombinant plasmids used for transfection were retransformed in *E. coli* SURE cells and grown in 200 ml TB-amp cultures of which plasmids were recovered by the maxiprep protocol (Green and Sambrook, 2012). The knockout plasmid was constructed using pHHTK as a base vector (Duraisingh et al., 2002). As 5′ and 3′ homology regions flanking the hDHFR resistance cassette the above 3′ fragment was used. The 5′ fragment was created by PCR amplification of a 735 bp-fragment using the oligonucleotides forward 5′-ACTAGTGCCAAGATACTGTTATTGTTGATGT and reverse AGATCTCTTCCACCTTACCGCTATTCC. The amplified fragment was cloned in pGEM T easy, Sanger-sequenced and subcloned *via* SpeI and BglII in the corresponding sites in pHHTK, resulting in pHHTK-5′AP2-O. Then, the 3′ gene fragment from the pGEM clone described above was excised with EcoRI and subcloned in pHHTK-5′AP2-O. After checking for orientation of the insert, the resulting vector pHHTK-AP2-O-KO was grown out in higher quantities and transfected as described below (see also **Supplementary Figure 1B**).

### Transfection of Blood Stage Parasites

Empty erythrocytes were electroporated with 40 µg of maxiprep-purified plasmid in incomplete cytomix following the Hasenkamp protocol (Hasenkamp et al., 2012) with slight modifications. 2 * 10^7^ mature parasites (schizonts) were concentrated up to 80% parasitemia by Voluven floating and mixed with the electroporated erythrocytes. After transfection, culture media were changed daily and parasites were submitted to drug pressure with 2.5 nM WR99210 (a gift from Jacobus Inc, USA) 48 h after transfection. Transfected parasites had their medium changed daily until day 6 when no more live parasites were visible. Once parasites reappeared (normally 16–25 days after transfection) and ring stage parasitemia reached 3–4%, several aliquots were frozen as backups. WR99210-resistant parasites were then cultivated for 20 days without WR99210 to promote the loss of episomal plasmids in parasites that did not recombine in the target locus. Then, WR99210 was re-added until parasitemia increased again and new aliquots were frozen. These drug on-off cycles were repeated three times to enrich recombinant parasites. Then, parasites were cloned by limiting dilution, and DNA was analyzed in order to identify clonal populations with the PfAP2-O locus modified using the oligonucleotides PfAP2-O “check” 5′-CATCAAAATGGATTTAATAATTGTTC and HA rev 5′-AGCGGCATAATCTGGAACATCGTAC.

### RNA Purification and cDNA Synthesis

Total RNA was converted to cDNA following the protocol described in (Gölnitz et al., 2008). When parasitemias reached 5%, RNA from parasites with and without Shield-1 treatment were harvested using Trizol (Life Technologies) following the manufacturer’s instructions. The final RNA pellet was resuspended in nuclease free water. For reverse transcription, 10 μg of total RNA was digested three consecutive times by the enzyme DNAse I (Fermentas). The material obtained from this reaction was used for cDNA synthesis using RevertAid reverse transcriptase (Fermentas) using random hexamer oligonucleotides (N_6_). *var* genes’ transcript amounts were measured using the primers reported in Salanti et al. (2003). The relative transcript quantities of different genes (see a list of oligos used for RT-PCR in **Supplementary Table 3**) were then calculated by the 2^−ΔCt^ method (Livak and Schmittgen, 2001) using the seryl tRNA ligase transcript as an endogenous control.

### Pull-down of proteins associated with AP2-O_GFP_HA_DD24

A total protein extract from the NF54::AP2-O_GFP_HA_DD24 clone was prepared and the protein complex containing the HA tag was purified using the HA-Tag (C29F4) Rabbit mAb (Sepharose Beads conjugate, Cell Signaling Technology) kit. The obtained eluate was separated in a 12% SDS gel using immunoprecipitated NF54 wild-type parasites as a control. To detect proteins in the precipitated complex, the SDS gel was submitted to silver staining using the PlusOne Silver Staining Kit (GE Healthcare, **Supplementary Figure 2**). We observed three different protein bands in the extract from the modified parasites compared to the negative control. These bands and bands corresponding to the same size in the negative control were excised, trypsin-digested, and analyzed by mass spectrometry. Analyses were performed on LTQ-Orbitrap Velos ETD (Thermo) coupled with Easy nanoLC II (Thermo). The peptides were separated on a C18RP column on a 95 min gradient. The instrumental conditions were checked using 50 fmol of a tryptic digest of BSA as standard. The sample carryover was completely removed between runs. The peptide search was conducted against the protein database *Plasmodium falciparum*_reviewed Uniprot. The complete list of proteins detected is shown in **Supplementary Table 2** and the analysis conditions are shown in **Supplementary Data File 2**.

### Adherent Phenotype Selection of Blood-Stage Parasites in Static Assays (Panning)

The selection of adhesive phenotypes of infected red blood cells (IRBCs) adherent to CHO-CD36 was done essentially as described in Gölnitz and colleagues (Gölnitz et al., 2008). Briefly, Voluven-floated trophozoite stage IRBCs (5 × 10^7^–10^8^, 50–80% parasitemia) were layered over confluent CHO-CD36 cells in RPMI/human plasma at pH6.8. Importantly, non-infected RBCs do not adhere to CHO-CD36 cells under these conditions [see also (Scherf et al., 1998; Gölnitz et al., 2008)]. The IRBCs were incubated over the cells for 1 h at 37°C and gently mixed every 15 min. After 1 h, the non-adherent IRBCs were aspired and the remaining adherent IRBCs were washed three times with RPMI at pH 6.8, and after the last washing step, the number of adherent IRBC was documented. The remaining IRBCs were then detached using RPMI/plasma at pH 7.2–7.4 and returned to the normal culture conditions. The process was repeated three times after which strongly adherent IRBCs predominantly expressing *var* gene PF3D7_0412400 (CHO-CD36 binding) were obtained. For the silencing assay, cells were grown in six-well plates, and central areas were marked in each well. For the cytoadherence readout, ten pictures for each condition were taken using the EVOS FL digital inverted microscope (AMG). The CHO cells and parasites were counted, and the results were plotted using GraphPad Prism 5 software.

### Fluorescence Microscopy

For GFP expression analysis, parasites were fixed as described in Tonkin et al. (2004) followed by incubation with PBS/Saponin 0.01% and DAPI at a final concentration of 2 µg/ml at 37°C for 1 h. After that, the parasites were washed three times with PBS/Saponin 0.01% and incubated with the membrane marker Wheat Germ agglutinin (WGA) Texas Red™-X conjugate (Invitrogen) in PBS/Saponin 0.01% for 20 min at 37°C. Finally, parasites were washed three times with PBS/Saponin. Images were acquired in a fluorescence microscope Zeiss Axio Observer Z1 and processed using Photoshop version 5.

### Nuclei Isolation and Fractionation

For the fractionation of nuclei, we followed the protocol described in Oehring et al. (2012). For this, parasites were first synchronized to detect PfAP2-O in each intraerythrocytic stage. Synchronized parasites were then lysed using saponin and washed three times with PBS. A second lysis was made using a hypotonic cytoplasmic lysis buffer CLB [20 mM HEPES (pH7.9), 10 mM KCl, 1 mM EDTA, 1 mM EGTA, 0.65% NP-40, 1 mM DTT, Complete protease inhibitors (Roche)] followed by centrifugation for 5 min at 2,000 g; the supernatant was used as the cytoplasmic fraction. Seven washes with the CLB buffer were then made followed by the nuclei extraction with low salt buffer LSB [20 mM HEPES (pH7.9), 0.1 M KCl, 1 mM EDTA, 1 mM EGTA, 1 mM DTT, protease inhibitors] during 20 min at 4°C. These extractions were used for western blot analysis.

### Western Blot

Protein extracts were separated in 10% SDS polyacrylamide gels and transferred to a Hybond C nitrocellulose membrane (GE Healthcare). After blocking with 4% non-fat milk in 1× PBS/0.1% Tween20, membranes were incubated with the murine antiHA (for PfAP2-O detection), the Rabbit-Anti TPK (*P. falciparum* Thiamin pyrophosphokinase) or mouse antiATC (Aspartate trans-carbamoylase, both cytoplasmic markers), and Rabbit-AntiH3 (Nuclei marker, Sigma-Aldrich, 1:1,000) or mouse anti-Pfs16 followed by antiRabbit or antiMouse IgG-peroxydase antibodies (KPL, 1:5,000) incubation. Blots were washed with PBS/Tween between incubations and finally incubated with WesternPico Super signal substrate (Pierce/Thermo) for detection. As a loading control, a murine polyclonal antiATC antibody (1:1,000) was used. The signal intensities were measured using the ImageJ program (NIH).

### *P. falciparum* Gametocyte Culture

*P. falciparum* wild type (NF54) and transgenic parasites PfAP2-O with and without Shield-1 were maintained under standard conditions to produce infectious gametocytes as described in Hyde et al. (2003). For this, two separate flasks of each strain were set up at 0.6% parasitemia and 6% hematocrit 14 and 17 days before the feeding using human blood obtained from the Scottish National Blood Transfusion Service. When parasitemia reached a high number and stressed parasites were observed in the blood smears the medium was increased by 50% and was changed every day until the day of mosquito feeding. Permission for the non-therapeutic use of human blood was obtained from the Scottish National Blood Transfusion Service Committee for the Governance of Blood and Tissue Samples for Non-Therapeutic Use, references 18–15.

### Mosquito Infection With *P. falciparum* Gametocytes

For each parasite line, 100 mosquitoes of *An. coluzzii* Ngousso strain were infected using membrane feeding (Ranford-Cartwright et al., 2016), with a mixture of day 14 and day 17 gametocyte cultures of each parasite line. Of note, the NF54::AP2-O-GFP-DD24 line was cultivated in the absence of Shield-1 during gametocyte induction. Gametocytes were centrifuged and the pellet resuspended in the same volume of human serum. Then, they were mixed with 1 to 2 volumes of 40% hematocrit human blood prepared in human serum, to produce a final gametocytemia of 0.5–1%. The gametocyte blood meal was placed into individual membrane feeders heated to 37°C, and mosquitoes were allowed to feed for 20–30 min. Unfed mosquitoes were removed, and the blood-fed mosquitoes were maintained under standard insectary conditions (26  ±  1°C, 80% humidity, 12 h light:12 h dark cycle) and fed *ad libitum* on 5% glucose solution containing 0.05% (w/v) 4-aminobenzoic acid (PABA). Mosquitoes were dissected 10 days post-infection, and the midguts were examined microscopically (400× magnification) for the presence and number of oocysts. Permission for the non-therapeutic use of human blood was obtained from the Scottish National Blood Transfusion Service Committee for the Governance of Blood and Tissue Samples for Non-Therapeutic Use, references 18–15. This experiment was done three times.

### Statistical Analysis of Mosquito Infection

To estimate the effect of the PfApiAP2-O knockout/knockdown in *P. falciparum* transmission, a generalized linear mixed-effect model GLMM was employed using the package lme4 (Bates et al., 2015) in R software version 3.5.3 (The R Foundation, 2013). To assess the effect on infection prevalence, a binomial GLMM with log link was used with fixed variables of parasite clone and gametocyte number present in the blood meal, with the replicate (infectious feed number) included as a random effect. A stepwise backward selection was used to identify the significant fixed effects (Burnham and Anderson, 2002), with optimal model selection performed using the Akaike Information Criterion (AIC) for which lower scores indicate better model fits, and likelihood ratio tests were used to compare the different models. Model diagnostics, including normality of residuals were done by visual inspection of residuals versus fitted values for each random effect group, and a Shapiro-Wilk test. Finally, to calculate how much of the variance was explained by the variables included in the final model, an R squared for the generalized linear mixed effect model r2glmm (Nakagawa et al., 2017) was applied. For this, the package MuMIn, version 1.41.0, was used, and the R2m indicates the variation in prevalence explained by the fixed effects, and the R2c included all the variables used in the model.

For intensity of infection (model 2), a zero-inflated negative binomial distribution was fitted to the data in a GLMM analysis using the package glmmTMB (Brooks et al., 2017) with the family nbinom 2 (log link). The final minimum model included fixed variables of clone and the number of gametocytes present in the blood meal, with feed number as a random effect: For model selection, diagnostics and to test overdispersion the same procedures from model 1 were used. As above, R software version 3.5.3 (R: The R Project for Statistical Computing) was used for data analysis and models fit, as additional packages were used lme4 (Bates et al., 2015), DHARMa 0.3.0 (Hartig, 2021) aods3 (Lesnoff et al., 2018) and MuMIn (v1.42.5) (Nakagawa et al., 2017).

## Results

### Decreased Quantities of PfAP2-O Do Not Interfere With *P. falciparum*Blood-Stage Growth

**Figure 2:**
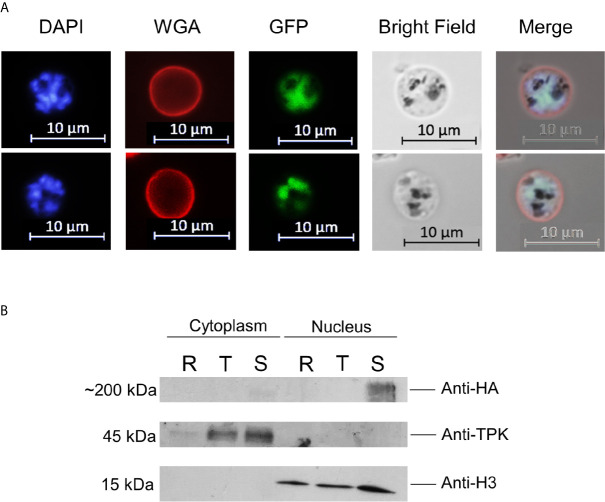
PfAP2-O-GFP-HA-DD24 is expressed in schizont stage parasites. **(A)** Fluorescence microscopy of late schizont parasites shows that GFP-tagged PfAP2-O colocalizes with the DAPI (nuclear stain) signal. WGA marks the surface of red blood cells. **(B)** Western blot of cytoplasmic and nuclear fractions obtained from PfAP2-O-GFP-HA-DD24 expressing parasites shows that AP2-O-GFP-HA-DD24 is found exclusively in the nucleus of schizonts. The membrane containing the transferred proteins was cut in three parts and was probed with monoclonal anti-HA (upper part), polyclonal anti-TPK (middle part), and monoclonal anti-Histone 3 antibodies (lower part). See **Supplementary Figures** for the full-size exposure.

NF54 parasites were successfully modified by single crossover recombination to contain a destabilizing DD24 domain (de Azevedo et al., 2012) in the 3′-position of the *PfAP2-O* open reading frame (**Figures 1A, B** and **Supplementary Figure S1**). Removal of Shield-1 from the cultures led to an ~85% depletion of the corresponding protein in mutant parasites (**Figure 1C** and **Supplementary Table 1**). Similar to knockout experiments of the orthologous gene in *P. berghei* (Yuda et al., 2009), a significant decrease in the expression levels of this factor did not result in a lethal growth defect after 96 h in the absence of Shield-1 compared to control parasites (**Figure 1D**). Considering that a severe growth defect in piggy-bac insertion mutants of PfAP2-O (Zhang et al., 2018) was observed, we tried to delete the gene to confirm a deleterious phenotype. After several attempts, we were not able to obtain mutant parasites using the classic approach described by Duraisingh et al. [**Supplementary Figure S1** (Duraisingh et al., 2002)]. This indicates that PfAP2-O is essential for blood stages but that even small amounts PfAP2-O under DD24 mediated knockdown are sufficient to maintain asexual growth. Using the GFP and HA tags contained in the transgenic strain, we observed a stage-specific expression and exclusive nuclear localization of the protein by a combination of fluorescent microscopy and nuclei fractionation assays (**Figure 2**). Of note, while PfAP2-O was only detected in the nuclear fraction of schizonts, Histone 3 was present in the ring, trophozoite, and schizont stage parasites. In contrast, Thiamin pyrophospho-kinase was visible exclusively in the cytosolic fraction of trophozoites and schizonts (**Figure 2**).

### PfAP2-O Knockdown Leads to Diversified Transcription Number of *var* Genes

**Figure 3:**
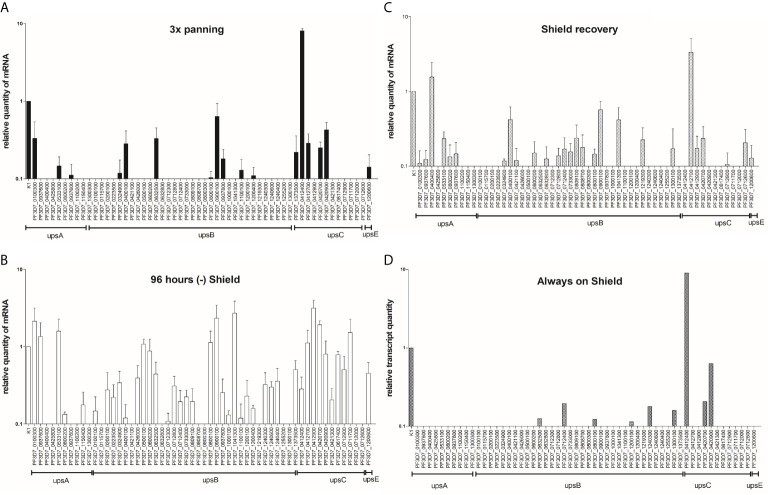
*var* gene transcription analysis during a transient *knockdown* in PfAP2-O-GFP-HA-DD24 parasites. **(A)** RNAs extracted from NF54::PfAP2-O-GFP-DD24 parasites grown in the presence of Shield-1, and panned three times over CHO-CD36 cells were analyzed by RT-qPCR using t-seryl ligase (“K1”) as an internal control transcript, as described in *Methods*. **(B)** *var* transcript profile after 96 h incubation of parasites without Shield-1. **(C)** *var* transcript profile in parasites after re-establishment of PfAP2-O for two reinvasions (96 h). **(D)** *var* transcription profile in parasites never submitted to silencing of PfAP2-O (Always on Shield-1, at the end of the knockdown experiment). The data represent results from three independent experiments and error bars indicate the standard deviation between these.

In a previous study, the single AP2 domain of PfAP2-O was predicted to bind to elements found in the 5′ ups regions of *var* genes (Campbell et al., 2010). To elucidate any influence of PfAP2-O on *var* transcription, we selected clonal parasite populations to dominantly express a single *var* gene (*PF3D7_0412400*) by repeated panning of PfAP2-O-GFP-DD24 expressing trophozoite/schizont-infected RBCs over CHO-CD36 cells, as described previously (Gölnitz et al., 2008) (**Figure 3A**). After this, we performed the silencing of the PfAP2-O for 96 h, followed by re-establishment of the protein to observe *var* gene transcripts during knockdown and after the reestablishment of PfAP2-O. *var* genes are exclusively transcribed during the first 10–14 h after merozoite invasion. Therefore, RNA samples from synchronized ring stage-parasites were collected from three differently treated cultures: 1. RNA from panned parasites (initial sample), 2. RNA from parasites with PfAP2-O knockdown by the absence of Shield-1 for two reinvasions, and 3. RNA from parasites two reinvasions after the knockdown and reestablishment of PfAP2-O by addition of Shield-1 (**Figure 3**).

In the absence of Shield-1, several var transcripts reproducibly showed increased relative amounts (*PF3D7_0100300, PF3D7_0412900, PF3D7_0420700, PF3D7_0500100, PF3D7_0533100, PF3D7_0711700, PF3D7_0800100, PF3D7_0900100, PF3D7_0937600, PF3D7_1041300*), while the initially dominant var gene transcript *PF3D7_0412400* was less abundant (**Figure 3B**). When checking for the chromosomal location of the differently transcribed var genes, we observed transcripts from all var subgroups, upsA, upsB, and upsC. Furthermore, while most of the upregulated var genes showed the 5′ ups recognition motifs identified for subdomains of PfAP2-O (Campbell et al., 2010), others did not, such as *PF3D7_0937600* and *PF3D7_0800100*. It appears that the depletion of PfAP2-O exerts mostly a de-repressing effect on var loci that were not exclusively related to any var gene subgroup or the predicted binding sites described by Campbell et al. (2010). Importantly, the continuous Shield-1 treatment itself does not alter var transcription or var transcription memory, since Shield-1-treated transfectant parasites used as control during these experiments showed similar var transcription patterns after selection on CHO-CD36 cells (**Figure 3D**).

### Short-Term Silencing of PfAP2-O Leads to the Complete Deletion of *var* Transcription Memory

The *var* transcription memory over multiple reinvasions is believed to be maintained by factors that direct chromatin readers and writers to their respective sites of action. Transcriptional activity of genes is reversibly determined in the specific histone code of methylation and acetylation of histone 3 lysines and probably others (Lopez-Rubio et al., 2007). If PfAP2-O is acting hierarchically upstream in the events that result in the recruiting of silencing and activating factors, then its knockdown should erase the epigenetic memory of *var* gene transcription. To verify what influence a temporary knockdown of PfAP2-O had on *var* transcription, we observed the *var* transcript profile when the protein function was re-established by re-adding Shield-1. The pre-silencing most active *var* locus *PF3D7_0412400* appeared to be silenced after the reestablishment of PfAP2-O. Instead, transcripts from its neighboring locus *PF3D7_0412700* and upsA *PF3D7_0400400* were dominantly detected, indicating that the transcriptional *var* memory had been erased. Again, cytoadherence-selected parasites that were cultivated on Shield-1 throughout the experiment were still dominantly expressing *var* gene *PF3D7_0412400*, confirming that no spurious global switching event occurred during the growth period (**Figures 3C, D**). This indicates that solely the retrieval and subsequent lower quantity of PfAP2-O accounted for the observed effect.

### The Absence of PfAP2-O Modifies the Cytoadherence Phenotype

**Figure 4:**
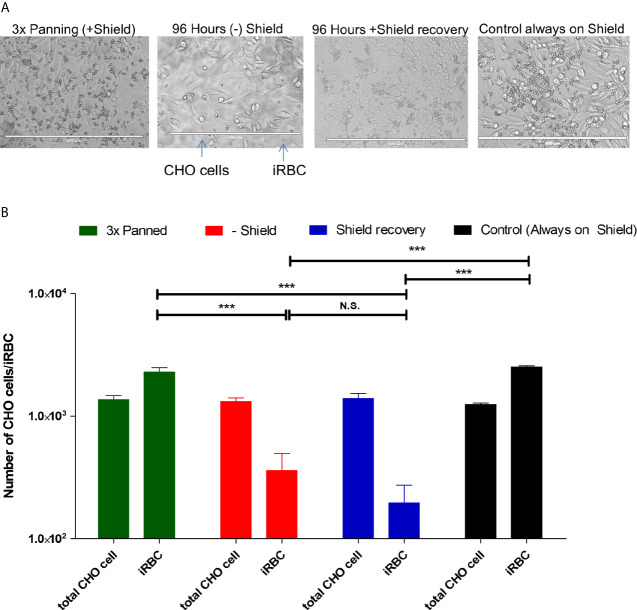
Adherent phenotype analysis shows decreased cytoadherence of IRBC during and after knockdown. **(A)** Illustrative recording of each condition. From these pictures, cells and the infected red blood cells were counted. The white bar at the bottom of each picture indicates 400 µm. **(B)** Graphic representation of three independent experiments. The significance of difference in numbers of adhering iRBC was calculated using Student’s T-Test, and three asterisks indicate *p* values <0.005. N.S. is not significant. “Controls” are the 3× CHO-CD36 selected cultures that were cultivated with Shield-1 throughout the test and analyzed at the same timepoint as the knocked-down and Shield-1 re-added cultures.

A landmark of different types of severe malaria is the adherence of infected red blood cells (IRBC) to determined receptors. Considering the observation that the PF3D7_0412400 *var* transcript was much less abundant in a transient knockdown of PfAP2-O, we tested if the parasites also lost the CHO-CD36 adherent phenotype. For this, a static cytoadherence procedure was conducted during and after the knockdown. Both CHO-CD36 cells and parasites were counted (**Figure 4**). We observed that during and after silencing of PfAP2-O the number of parasites adhering to CHO-CD36 cells was significantly lower than the initially CHO-CD36 selected parasites and also the control parasite line, indicating that this factor may influence *P. falciparum* cytoadherence mediated by PfEMP1 proteins.

### PfAP2-O Seems to Interact With Histone Modifiers

PfAP2-O probably interacts with other factors to exert its biological role. To identify proteins that physically interact with PfAP2-O, a total protein extract from schizonts was prepared and PfAP2-O was immunoprecipitated *via* the HA tag and purified. Three protein bands at molecular weights of 70, 30, and 15 kDa (which did not appear in NF54 wildtype extracts immunoprecipitated in parallel) were detected and analyzed by mass spectrometry (**Supplementary Figure S2**). In all of these extracts, fragments of a 167 kDa protein appeared, and this protein (PF3D7_0216700/PFB0765w) was predicted to be related to autophagy. Fragments of an essential 402 kDa protein with unknown function (PF3D7_0317300/PFC0650w) were detected in the 70 kDa and the 15 kDa fractions. We also observed that PfAP2-O associated with two histone modifiers namely histone-lysine N-methyltransferases SET1 (encoded by *PF3D7_0629700*) and SET2 (encoded by *PF3D7_1322100*, PfSET2/PfSET-VS) and another member of the ApiAP2 family (*PF3D7_0420300*, **Supplementary Table 2**). Intriguingly, no peptides from histones were identified in this assay, indicating that PfAP2-O may not directly interact with these.

### The Role of PfAP2-O in *P. falciparum* Sexual Development

Several members of the ApiAP2 family have been associated with important processes in the *Plasmodium* life cycle, such as gametocyte commitment (*PfAP2-G/PfAP2-G2*) (Kafsack et al., 2014; Yuda et al., 2015), liver stage (Iwanaga et al., 2012), sporozoite (*PbAP2-SP*) (Yuda et al., 2010), and oocyst (*PbAP2-O*) (Yuda et al., 2009) development, and invasion ligand transcription (*PfAP2-I*) (Santos et al., 2017). Considering this, we tested if the knockdown of PfAP2-O regulated any gametocyte-related transcripts induced in the presence of PfAP2-G as well as the transcript of PfAP2-G itself.

**Figure 5:**
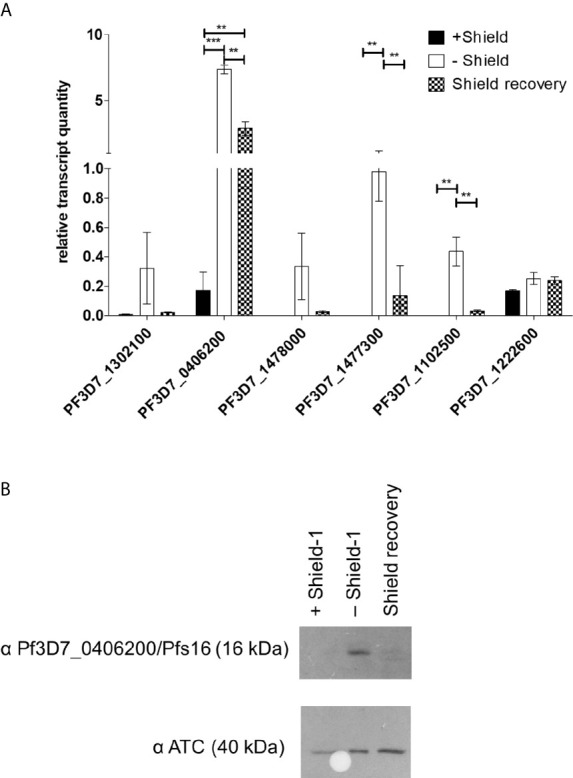
PfAP2-O-GFP-HA-DD24 is involved in the transcription/translation of gametocyte-related genes in *P. falciparum*. **(A)** Transcript abundance of gametocyte-stage specific genes in PfAP2-O-GFP-HA-DD24 expressing, knocked-down (two reinvasions without Shield-1), and recovered parasites (two reinvasion cycles without Shield-1, followed by two reinvasion cycles in the presence of Shield-1). The data were normalized to seryl-tRNA ligase (PF3D7_0717700), used as the internal control. For statistical evaluation, the ANOVA test with Bonferroni’s correction was used. ** is p < 0.01 and *** is p < 0.005. **(B)** Western blot detecting Pfs16/Pf3D7_0406200 in extracts of NF54::PfAP2-O_GFP_HA_DD24 parasites in the three conditions (on Shield-1/knocked-down/recovered parasites). As a control, an anti-ATC antibody was used as before. See **Supplementary Figures** for the full size exposure.

The PfAP2-O silencing had a mixed effect on gametocyte-associated transcripts in trophozoite stage parasites. While no difference was discernible in the steady-state transcript quantities of PfAP2-G (*PF3D7_1222600*), several key gene transcripts previously associated with gametocyte development appeared in increased amounts (**Figure 5A**). The transcript of the early gametocyte-expressed gene *Pfs16* (*Pf3D7_0406200*) appeared to increase up to four-fold when compared with the control. When the PfAP2-O protein was re-established, we observed that most of the transcripts returned to previous expression levels, except for Pfs16, which remained at high levels. To investigate if the Pfs16 transcript was indeed being translated, its corresponding protein was detected with a specific antibody. While during PfAP2-O-GFP knockdown Pfs16 was readily detected, re-establishment of PfAP2-O-GFP (**Figure 5B**) resulted in the disappearance of the protein.

### PfAP2-O Is Essential for *P. falciparum* Sexual Development

**Figure 6:**
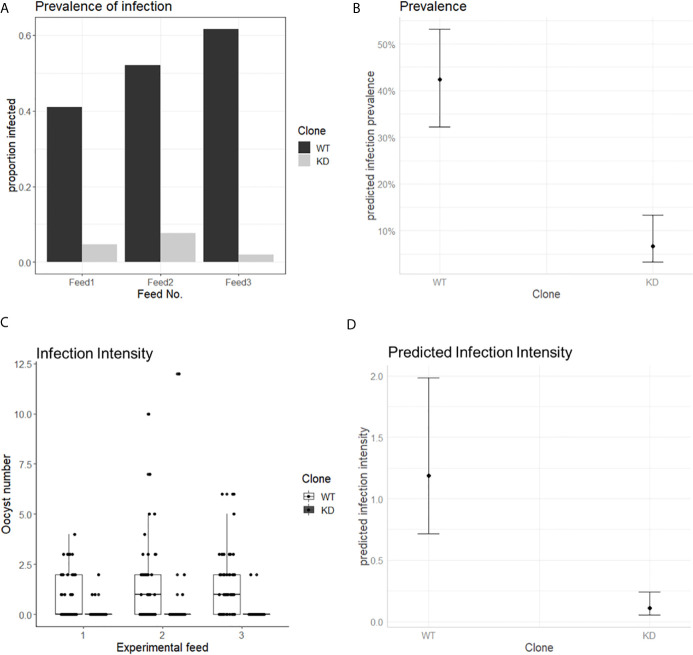
PfAP2-O is essential for *P. falciparum* transmission to *Anopheles coluzzii*. Mosquitoes were fed with gametocytes of NF54 and NF54::AP2-O-GFP-DD24, induced in the absence of Shield-1 (see *Methods* and **Supplementary Figure 3** for details). **(A)** Prevalence of infected mosquitoes fed with ApiAP2-O *knockdown* and WT gametocytes in each experimental feeding. **(B)** Predicted effect of parasite clone on mosquito infection, bars indicate 95% confidence intervals. **(C)** number of oocysts observed in each experimental feed. **(D)** Predicted effect of each clone in oocyst number. Bars indicate 95% confidence intervals.

The murine *P. berghei* AP2-O protein (PbApiAP2-O) was associated with mosquito transmission since knockout parasites for this protein could not form functional ookinetes capable of invading the *Anopheles* midgut and thus completing the sporogonic cycle (Yuda et al., 2009). We then asked whether the PfAP2-O protein would have the same effect in *P. falciparum*. For this, *Anopheles coluzzii* mosquitoes were fed with the PfAP2-O-GFP-DD24 strain in the absence of Shield-1 (AP2-O *knockdown*, KD), and the prevalence of infected mosquitoes and oocyst number was measured and compared with the NF54 WT strain. In all experimental feedings, the infection prevalence was lower than 10% for the KD strain and higher than 40% for the WT (**Figure 6A**). Using a generalized linear mixed model (GLMM) (**Supplementary File 1**) to predict the effects of PfAP2-O knockdown on parasite transmission, we observed a significantly lower oocyst prevalence in mosquitoes infected with the thePfAP2-O knockdown line compared to the NF54 wild type (**Figure 6B**, P-value: 2.6 * 10^−6^), controlling for gametocyte numbers in the infectious blood meal which was positively correlated with infection (P = 0.0158). The odds ratio values indicated that mosquitoes fed blood meals containing knockdown parasites had a significantly lower probability of infection than those fed with the WT parasites (OR: 0.09, 95% CI). We also observed that a higher number of gametocytes in the infectious blood meal correlated with increased chances of infection (OR: 1.56, 95% CI).

When the intensity of the infection (oocyst numbers) in mosquitoes was analyzed, we observed that in all experiments, the number of oocysts in mosquitoes fed with KD parasites was significantly lower compared to those fed with the NF54 WT parasites (**Figure 6C**). In a GLMM analysis, infection intensity (**Supplementary File 1**) was significantly influenced by the parasite clone used (P = 5.61 * 10^−14^), but not by the gametocyte number in the blood meal (P: 0.0541) (**Figure 6D**).

These data show that the knockdown of PfApiAP2-O significantly decreased both the prevalence and the intensity of infection in mosquitoes and predict a positive relation between the expression of the protein and the transmission of *P. falciparum* from human to *Anopheles* mosquitoes.

## Discussion

Transcriptional regulation in *Plasmodium falciparum* is typically related to chromatin modification and in this regard, histone methyltransferases, demethylases, a histone acetyltransferase, and deacetylases have a central role (Tonkin et al., 2009). These factors are believed to regulate the accessibility of transcription factors to promoter regions [revised in (Hollin et al., 2021)]. In the case of *var* genes, however, the hierarchy of events that finally leads to the selective activation of one promoter and the inactivation of the previously active one (switching) is unclear. We hypothesized that by using a temporary knockdown of factors putatively participating in these events, the hierarchy of elements playing a role in the maintenance or modification of epigenetic signatures of *var* transcription might be elucidated.

Members of the ApiAP2 TFs family seem to be key regulators in the *Plasmodium* life cycle since some of them have been associated with important processes such as transcription control of multigenic families, gametocyte commitment, and invasion of erythrocytes in *P. falciparum* (Kafsack et al., 2014; Martins et al., 2017; Santos et al., 2017), oocyst, and sporozoite formation in *P. Berghei* (Yuda et al., 2009; Yuda et al., 2010), and liver stage development also in *P. Berghei* (Iwanaga et al., 2012). Here we explored the function of PfApiAP2-O in *P. falciparum* asexual and sexual cycles with a particular focus on the control of *var* gene transcription. We successfully modified the target gene with various sequences that permitted to examine its function in different molecular and biological processes of the parasite life cycle. We observed a stage-specific expression and a nuclear localization through the GFP/HA tags, which is expected for DNA binding proteins. The knockdown assays showed that partial depletion of this factor has not a lethal effect on the asexual proliferation as we did not observe any defect or delay in parasite growth when the protein was absent.

In the murine malaria *P. berghei*, PbAP2-O has a crucial role in ookinete formation and invasion of mosquito midgut with no essential function during the intraerythrocytic cycle (Yuda et al., 2009). Similarly, normal levels of PfAP2-O seemed also not essential for asexual proliferation in *P. falciparum*, and long-term growth in the absence of Shield-1 revealed no change in parasite development or growth rate during the asexual intraerythrocytic cycle. However, several attempts to fully disrupt the *PfAP2-O* gene failed in our hands. Along with the growth defect shown in insertion-mutated versions of this protein showed by Zhang et al. (2018), these observations point to a lethal phenotype when this factor is completely absent during the intraerythrocytic cycle. As we observed that the knockdown was not complete, we hypothesize that the few functional PfAP2-O were still sufficient to maintain normal asexual growth. Moreover, we observed that PfAP2-O, in contrast to PbAP2-O (Yuda et al., 2009), is expressed in schizont stage parasites, already indicating additional roles besides the control of expression of female gametocyte- and ookinete-related genes.

The knockdown of PfAP2-O led to a swift and complete change of *var* transcription patterns, indicating that PfAP2-O is decisively involved in maintaining the transcriptional memory and acts as an important factor in *var* switching. Based on the results of previous works (Chookajorn et al., 2007; Lopez-Rubio et al., 2007; Salcedo-Amaya et al., 2009), we hypothesize that histone lysine 9 modifications (trimethylation or acetylation) are different at *var* loci before and after knockdown of PfAP2-O. In consequence, PfAP2-O seems to guide chromatin-modifying factors to silenced loci, since in the absence of PfAP2-O, several *var* loci—but not all—were actively transcribed. This is enforced by the fact that different histone modifiers were identified in the proteomic analysis. The transcript analysis also revealed that the same *var* loci were activated in three biological replicates. Thus, it appears that parasites with the activated *var* locus Pf3D7_0412400 preferentially switch to the same adjacent *var* (PF3D7_0412700 in this case) when PfAP2-O is temporarily unavailable. Perhaps small non-coding RNAs from the *var* locus-adjacent RUF6 loci are involved in this phenomenon (Wei et al., 2015; Fan et al., 2020).

The change of *var* transcription was also reflected in the change of cytoadherence patterns in parasites subjected to PfAP2-O protein depletion. Here, IRBC under PfAP2-O knockdown adhered significantly less to CHO-CD36 cells, probably due to the virtual disappearance of the PfEMP1 that most effectively binds CD36, encoded by Pf3D7_0412400. Taking into account that IRBC binding to CHO-CD36 occurred in fact *via* CD36 and not *via* other unrelated receptors found on CHO cells (Andrews et al., 2005), then interaction with the surface of CHO-CD36 cells with the PfEMP1 ectodomain present in Pf3D7_0412400 is stronger than that of the PF3D7_0412700. Both PfEMP1 ectodomains were predicted to be competent for CD36 binding, while the second most transcribed *var* PF3D7_0400400 encodes a CIDR*α*1.1 domain, which is not expected to interact with CD36 (Hsieh et al., 2016). The *var* PF3D7_0400400, upregulated after knockdown and recovery of PfAP2-O, is the so-called *var_severe_* (Lavstsen et al., 2012) of the upsA *var* group of which expression is upregulated in severe malaria cases (Warimwe et al., 2009) and the onset of infection in non-immune individuals (Lavstsen et al., 2005). Another explanation of decreased cytoadherence under/after AP2-O knockdown may be that knob-associated proteins such as KAHRP or PfEMP3 were also influenced. In endemic areas, the disruption of cytoadherence remains a challenge in the treatment of patients with severe malaria (Mustaffa et al., 2017). The fact that the plant-like ApiAP2 proteins are not encoded in the human host may point PfAP2-O to a novel target of intervention to block the adhesion of infected red blood cells, perhaps alleviating life-threatening cytoadherence patterns.

Histone protein 1 (HP-1) has previously been shown to control transcription of gametocyte-associated genes by de-repression of PfAP2-G, but also that of variant genes such as *var, rif*, and *Pfmc-2TM* (Brancucci et al., 2014). Here, we demonstrated a repressive effect of the PfAP2-O factor, which may be independent of HP1, since no tight interaction between PfAP2-O and HP1 was observed by our proteomic analysis. Based on the upregulation of *var* and some gametocyte-associated genes when the PfAP2-O is depleted, we hypothesize that PfAP2-O indirectly triggers a repressive effect on its target genes. PfAP2-O may promote the recruitment of HP-1 to specific sites, which then leads to gene silencing; in the absence of PfAP2-O, the HP-1-guiding effect would then be impaired at least for some loci. The role of PfAP2-O on variant gene control is further supported by the observation that different histone modifiers such as PfSET1 and also PfSET2 co-precipitated with it and these have already been linked to *var* transcription (Jiang et al., 2013). Also, the factor identified as Pf08_0048, described as a putative Snf2-related CBP activator (chromatin remodeler) may be involved in *var* transcription, although the factor was not identified in the recent deadCas9-based approach to identify proteins associated to *var* loci (Bryant et al., 2020).

Regarding sexual commitment, we detected the increased presence of several early gametocyte-related transcripts in PfAP2-O-knocked-down parasites. Transcription of the early gametocyte gene Pfs16 (Pf3D7_0406200) was strongly increased upon PfAP2-O knockdown but did not return to pre-knockdown levels after re-exposure of the parasites to Shield-1. This may indicate the passing of a checkpoint (still undescribed) that leads to the persisting of Pfs16 transcription. However, despite the presence of Pfs16 transcript, Pfs16 protein was no longer detected after re-establishment of PfAP2-O, suggesting additional post-transcriptional control of this protein. Commitment to gametocyte development is known to be controlled by another member of the ApiAP2 family, PfAP2-G/PF3D7_1222600: Increases in PfAP2-G leads to increased transcription of gametocyte-related genes and conversion to gametocytes (Kafsack et al., 2014). The increased expression of PfAP2-G had no significant influence on the transcription of PfAP2-O (Kafsack et al., 2014), conversely, we observed that knockdown of PfAP2-O had no enhancing effect on the presence of the PfAP2-G transcript (**Figure 5A**).

We also investigated the role of PfAP2-O in parasite transmission through mosquito infection. There was no difference in the number of mature gametocytes obtained under standard culturing conditions for the WT and KD clones (**Supplementary Figure S3**). Furthermore, the gametocytes produced by the PfAP2-O-knocked-down line did not present any visible difference in shape or size compared to the NF54 (**Supplementary Figure 3**). Gametocytes produced under PfAP2-O knockdown were able to exflagellate, which indicates that this protein does not play an important role in gametocyte commitment and exflagellation. However, it is still uncertain whether these gametocytes have a biological defect that blocks the fertilization and mosquito midgut invasion, given that a number of gametocyte-related genes were deregulated (see **Figure 5**). When analyzing genomic features of the differentially regulated gametocyte-related genes, we found that all of them were centromeric, and only one gene (PF3D7_1477300, a PHIST gene) can be occupied by HP1, while the others (Pf3D7_1302100, Pf3D7_0406200, Pf3D7_ 1478000, Pf3D7_1102500) are not occupied by HP1 (Fraschka et al., 2018). This points to an additional layer of transcriptional control of these genes exerted directly or indirectly by AP2-O. In our experiments, we did not study fertilization and ookinete formation to assess this question due to the difficulties to induce them *in vitro* in *P. falciparum*. Our results showed that mosquitoes fed with the PfApiAP2-O strain under knockdown had a lower level of infection than those fed with the NF54 WT control, similar to the results shown for the PfAP2-O ortholog in *P. berghei* (Yuda et al., 2009). We showed that protein depletion of PfApiAP2-O led to a significant decrease in the prevalence of infected mosquitoes as well as a reduction of oocyst numbers. Thus, PfAP2-O appears to be essential for mosquito invasive stages, as was shown for its ortholog in *P. berghei*. These observations suggest a conservative role of this protein in human-mosquito transmission among *Plasmodium* species. Finally, the ApiAP2-O knockdown did not result in a complete blockade of mosquito infection: a minority of mosquitoes still was infected, with very low oocyst numbers. This could be explained by the fact that the knockdown was partial and small amounts of protein are still detected during Shield withdrawal.

Interactions between ApiAP2 proteins in *P. berghei* are involved in crucial processes during the parasite life cycle, including gametocyte development, and oocyst and sporozoite formation (Modrzynska et al., 2017). In our proteomic co-immunoprecipitation analysis another ApiAP2 member, PF3D7_0420300/PFD0985W, with no defined function, was also detected. Campbell and colleagues identified a significant number of target genes predicted for this protein, mainly associated with DNA replication and gene expression (Campbell et al., 2010). These observation suggests that these members may be part of a protein interaction network responsible for important steps in the *P. falciparum* life cycle, similar to that described in the murine parasite (Modrzynska et al., 2017).

In conclusion, we showed that PfAP2-O controls transcript levels of the *var* family, antigenic variation, and transcriptional memory in the *P. falciparum* asexual cycle. We also demonstrated a significant decrease in *P. falciparum* transmission to *Anopheles* mosquitos when this protein is absent. PfAP2-O apparently interacts with other transcription factors and histone modifiers in a dynamic protein complex involved in several key processes in *P. falciparum* biology. A further ChIPseq and RNAseq analyzing ring, trophozoite, and schizont stages may reveal further details about the interactors of AP2-O. Due to its influence on variant gene transcription and transmission, PfAP2-O may be an attractive drug target to alleviate virulence factor-induced pathogenesis, with additional impacts through blocking the passage of the parasite from an infected human to the invertebrate host.

## Data Availability Statement

The original contributions presented in the study are publicly available. This data can be found here: Wunderlich, Gerhard (2021), “DatasetsAP2-Oknockdown”, Mendeley Data, V1, doi: 10.17632/rrx9bsmyrv.1. The mass spectrometry proteomics data have been deposited to the ProteomeXchange Consortium via the PRIDE (Perez-Riverol et al., 2019) partner repository with the dataset identifier PXD026237.

## Author Contributions

EC, LR-C, and GW conceived the study. EC, IO, WF, LR-C, and GW conducted experiments. EC, LR-C, and GW analyzed the data and drafted the manuscript. All authors contributed to the article and approved the submitted version.

## Funding

Research in GW’s lab is funded by the Fundação de Apoio à Pesquisa do Estado de São Paulo (FAPESP, grants 2015/17174-7 and 2017/24267-7). EC and WF were supported by FAPESP fellowships (2016/12659-5 and 2016/19145-7, respectively), and IO was supported by a Conselho Nacional de Pesquisa (CNPq) fellowship. GW is a CNPq Research fellow.

## Conflict of Interest

The authors declare that the research was conducted in the absence of any commercial or financial relationships that could be construed as a potential conflict of interest.
