# Supplementary material for: The Transcription Factor PfAP2-O Influences Virulence Gene Transcription and Sexual Development in Plasmodium falciparum
Source: Front Cell Infect Microbiol. 2021 Jun 28;11:669088. doi: 10.3389/fcimb.2021.669088 (PMC8275450; doi:10.3389/fcimb.2021.669088)
Supplement: Supplementary file 1 [file DataSheet_1.pdf]

CEFAP USP
Centro de Facilidades de Apoio à Pesquisa
Universidade de São Paulo
USP

Fevereiro 2018

Analyses are performed on LTQ-Orbitrap Velos ETD (Thermo) coupled with Easy nanoLC II (Thermo). The peptides are separated on a C18RP column on a 95 min gradient. All the database searching parameters are attached below.
The instrumental conditions are checked using 50fmol of a tryptic digest of BSA as standard.
The sample carryover is completely removed between run.

The sample analyzed is named:

1) 15kDa
2) 30kDa
3) 70kDa

The search was made against protein database:
Plasmodium falciparum_reviewed Uniprot

The data is presented in 1 excel file named:

1) 15kDa.xls
2) 30kDa.xls
3) 70kDa.xls

The file contain the proteins grouped identified in the sample.
The file also contain the redundant peptides identified in the sample.
The file contain also the further parameters specific to each peptide.

.BIOMASS team

Search parameters:
1. Input Data:

Enzyme Name: Trypsin (Full)
Max. Missed Cleavage Sites: 2
Min. Peptide Length: 6
Max. Peptide Length: 144

2. Scoring Options:

Max. Delta Cn: 0.05
Max. Number of Peptides Reported: 10

3. Tolerances:

Precursor Mass Tolerance: 20 ppm
Fragment Mass Tolerance: 0.6 Da
Use Average Precursor Mass: False
Use Average Fragment Mass: False

4. Spectrum Matching:

Use Neutral Loss a Ions: True
Use Neutral Loss b Ions: True
Use Neutral Loss y Ions: True
Use Flanking Ions: True
Weight of a Ions: 0
Weight of b Ions: 1
Weight of c Ions: 0
Weight of x Ions: 0
Weight of y Ions: 1
Weight of z Ions: 0

5. Dynamic Modifications:

Max. Equal Modifications Per Peptide: 3
Max. Dynamic Modifications Per Peptide: 4
N-Terminal Modification: Acetyl / +42.011 Da (Any N-Terminus)
1. Dynamic Modification: Oxidation / +15.995 Da (M)

6. Static Modifications:
----
1. Static Modification: Carbamidomethyl / +57.021 Da (C)

Processing node 3: Percolator
----

1. Input Data:

Maximum Delta Cn: 0.05

2. Decoy Database Search:

Target FDR (Strict): 0.01
Target FDR (Relaxed): 0.05

Validation based on: q-Value
