# Supplementary material for: The Transcription Factor PfAP2-O Influences Virulence Gene Transcription and Sexual Development in Plasmodium falciparum
Source: Front Cell Infect Microbiol. 2021 Jun 28;11:669088. doi: 10.3389/fcimb.2021.669088 (PMC8275450; doi:10.3389/fcimb.2021.669088)
Supplement: Supplementary file 2 [file DataSheet_2.docx]

Supplementary Data:

GLMM model for infection prevalence (binomial model, logit link)

Fixed Variables: Parasite clone (WT or AP2-O knockout); gametocyte density in infectious blood meal.

Random variable: Experimental replicate

Estimate Std. Error z value Pr(>|z|)

Fixed effects:

(Intercept) -3.0526 1.3036 -2.342 0.0192 *

Clone -2.3270 0.4951 -4.700 2.6e-06 ***

Gametocyte density 0.4452 0.1845 2.413 0.0158 *

Significance codes: 0 '***' 0.001 '**' 0.01 '*' 0.05 '.' 0.1 ' ' 1

R^2^m=0.43; R^2^c=0.43

Model significance compared to null model (random variable only) X^2^=108.28, d.f.=2, p=2.2e^-16^.

GLMM model for infection Intensity (zero-inflated negative binomial logit link)

Fixed Variables: Parasite clone (WT or AP2-O knockout);

Random variable: Experimental replicate

Estimate Std. Error z value Pr(>|z|)

(Intercept) 0.5018 0.1824 2.752 0.00593 **
 CloneKD -2.8257 0.3759 -7.517 5.61e-14 ***

Signif. codes: 0 '***' 0.001 '**' 0.01 '*' 0.05 '.' 0.1 ' ' 1
