# Supplementary material for: The Transcription Factor PfAP2-O Influences Virulence Gene Transcription and Sexual Development in Plasmodium falciparum
Source: Front Cell Infect Microbiol. 2021 Jun 28;11:669088. doi: 10.3389/fcimb.2021.669088 (PMC8275450; doi:10.3389/fcimb.2021.669088)
Supplement: Supplementary file 3 [file Presentation_1.ppt]

## Slide 1
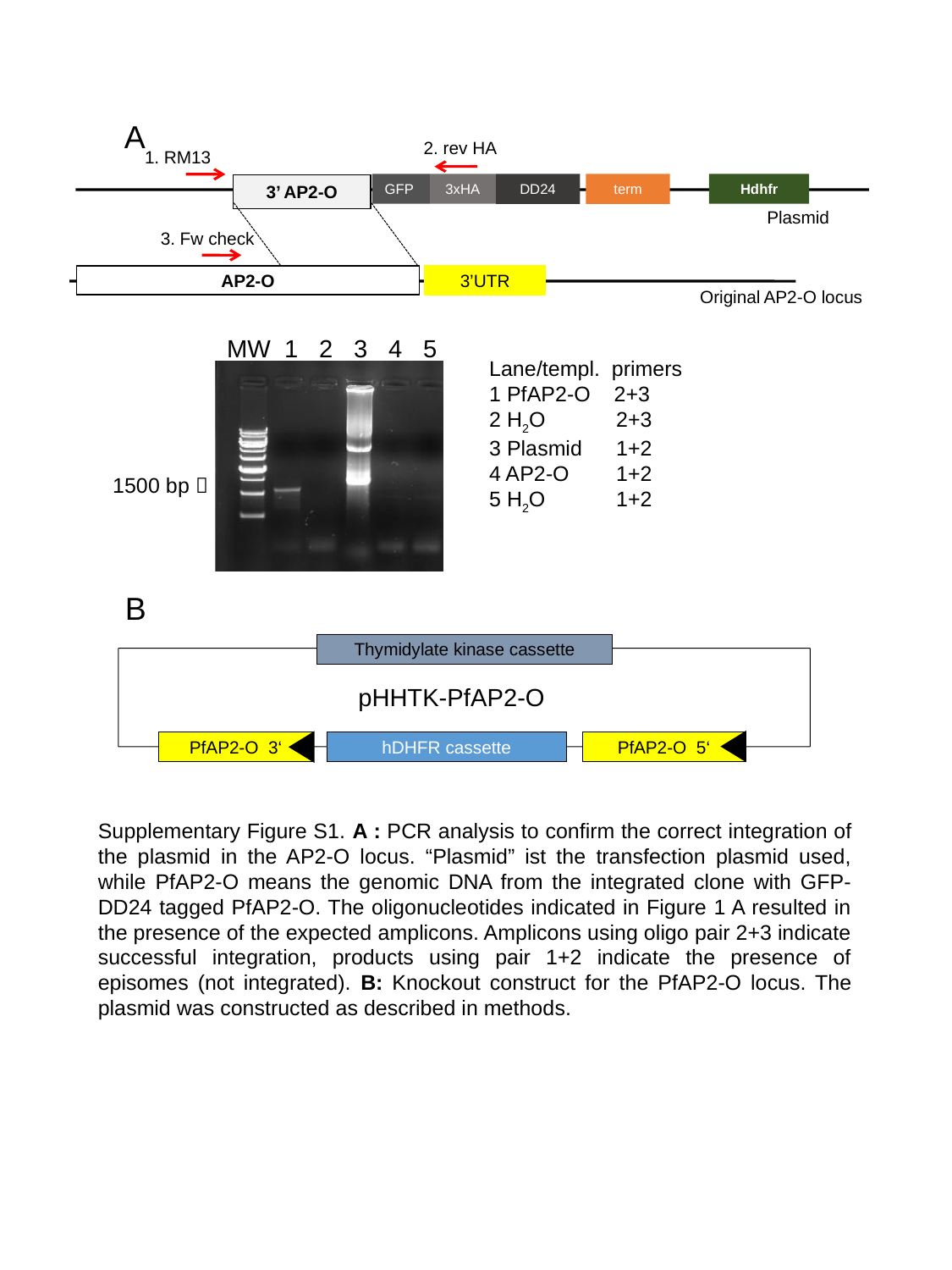

A
2. rev HA
1. RM13
GFP
3xHA
DD24
term
Hdhfr
3’ AP2-O
Plasmid
3. Fw check
AP2-O
3’UTR
Original AP2-O locus
 MW 1 2 3 4 5
1500 bp 
Lane/templ. primers
1 PfAP2-O 2+3
2 H2O 	2+3
3 Plasmid 	1+2
4 AP2-O	1+2
5 H2O 	1+2
B
Thymidylate kinase cassette
PfAP2-O 3‘
hDHFR cassette
PfAP2-O 5‘
pHHTK-PfAP2-O
Supplementary Figure S1. A : PCR analysis to confirm the correct integration of the plasmid in the AP2-O locus. “Plasmid” ist the transfection plasmid used, while PfAP2-O means the genomic DNA from the integrated clone with GFP-DD24 tagged PfAP2-O. The oligonucleotides indicated in Figure 1 A resulted in the presence of the expected amplicons. Amplicons using oligo pair 2+3 indicate successful integration, products using pair 1+2 indicate the presence of episomes (not integrated). B: Knockout construct for the PfAP2-O locus. The plasmid was constructed as described in methods.

## Slide 2
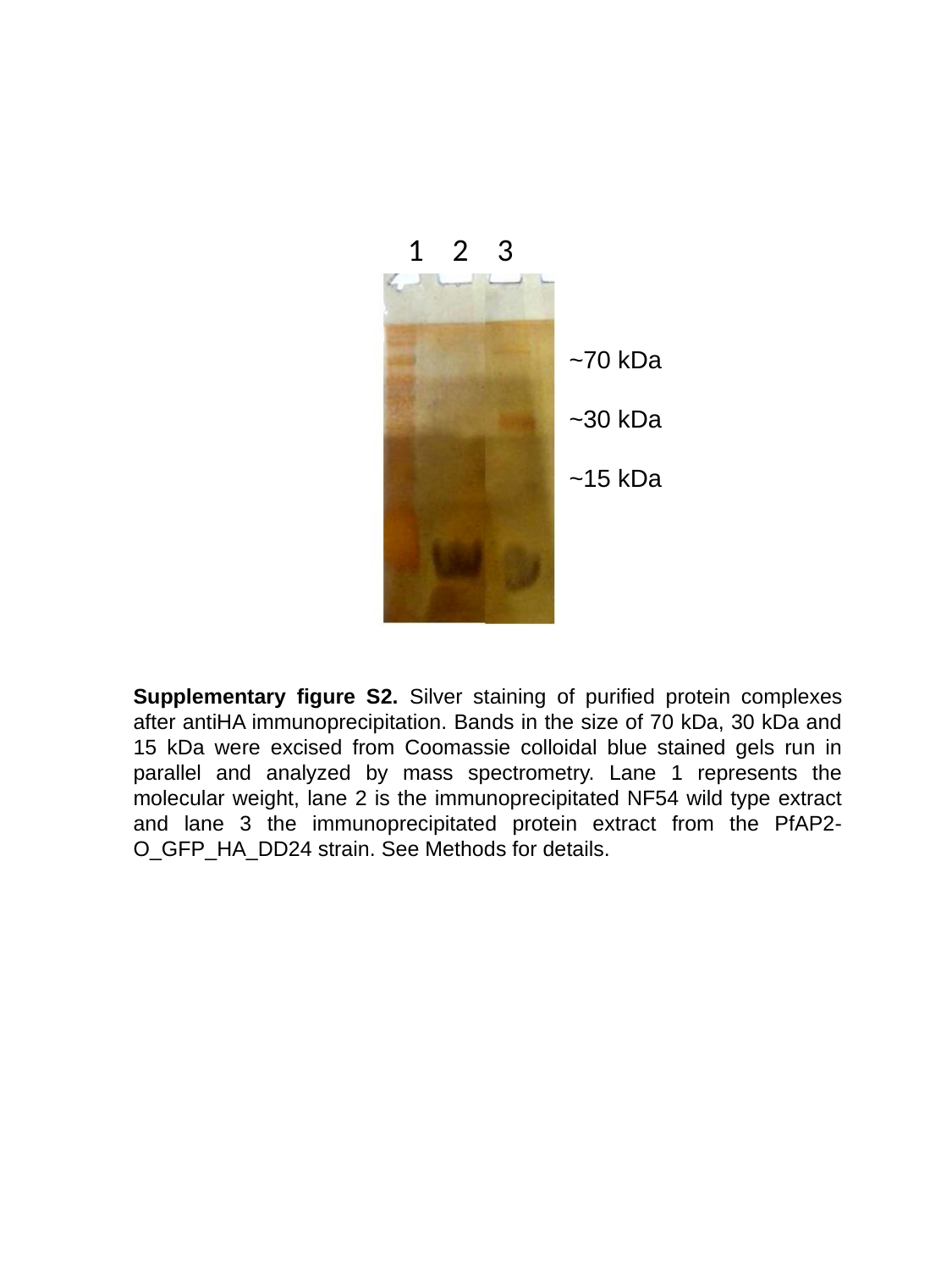

1 2 3
~70 kDa
~30 kDa
~15 kDa
Supplementary figure S2. Silver staining of purified protein complexes after antiHA immunoprecipitation. Bands in the size of 70 kDa, 30 kDa and 15 kDa were excised from Coomassie colloidal blue stained gels run in parallel and analyzed by mass spectrometry. Lane 1 represents the molecular weight, lane 2 is the immunoprecipitated NF54 wild type extract and lane 3 the immunoprecipitated protein extract from the PfAP2-O_GFP_HA_DD24 strain. See Methods for details.

## Slide 3
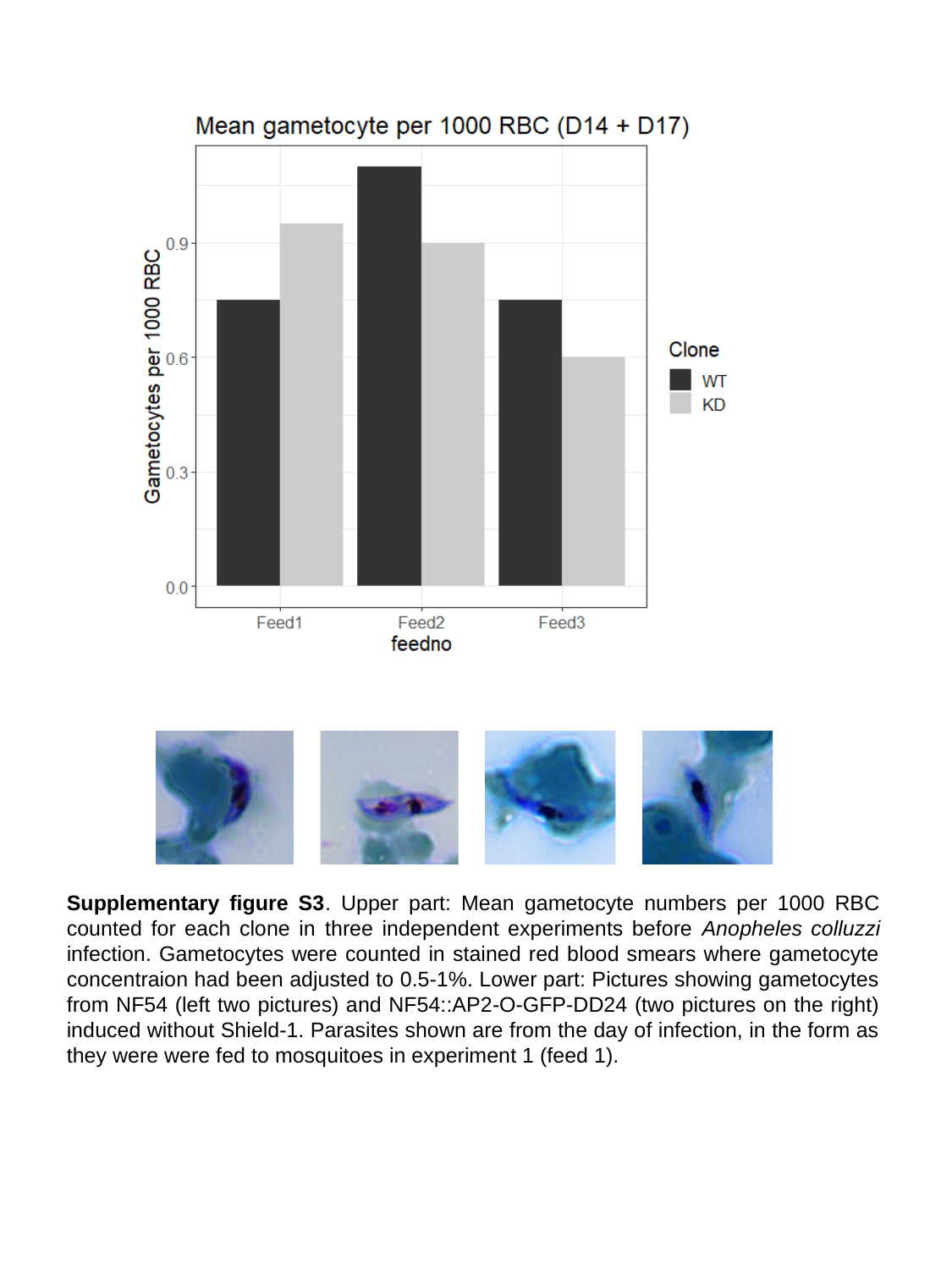

Supplementary figure S3. Upper part: Mean gametocyte numbers per 1000 RBC counted for each clone in three independent experiments before Anopheles colluzzi infection. Gametocytes were counted in stained red blood smears where gametocyte concentraion had been adjusted to 0.5-1%. Lower part: Pictures showing gametocytes from NF54 (left two pictures) and NF54::AP2-O-GFP-DD24 (two pictures on the right) induced without Shield-1. Parasites shown are from the day of infection, in the form as they were were fed to mosquitoes in experiment 1 (feed 1).

## Slide 4
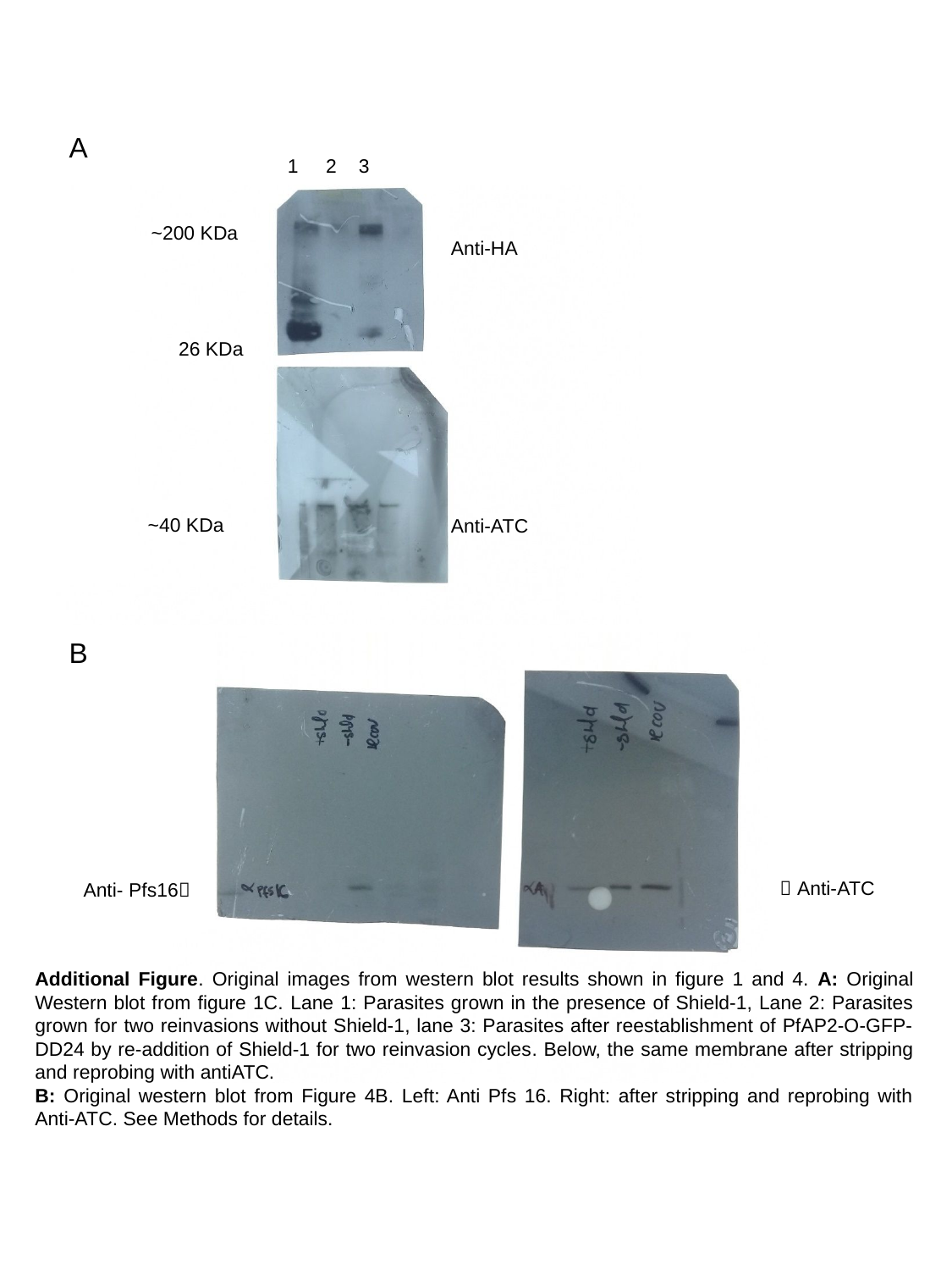

A
1 2 3
~200 KDa
 26 KDa
Anti-HA
Anti-ATC
~40 KDa
B
 Anti-ATC
Anti- Pfs16
Additional Figure. Original images from western blot results shown in figure 1 and 4. A: Original Western blot from figure 1C. Lane 1: Parasites grown in the presence of Shield-1, Lane 2: Parasites grown for two reinvasions without Shield-1, lane 3: Parasites after reestablishment of PfAP2-O-GFP-DD24 by re-addition of Shield-1 for two reinvasion cycles. Below, the same membrane after stripping and reprobing with antiATC.
B: Original western blot from Figure 4B. Left: Anti Pfs 16. Right: after stripping and reprobing with Anti-ATC. See Methods for details.

## Slide 5
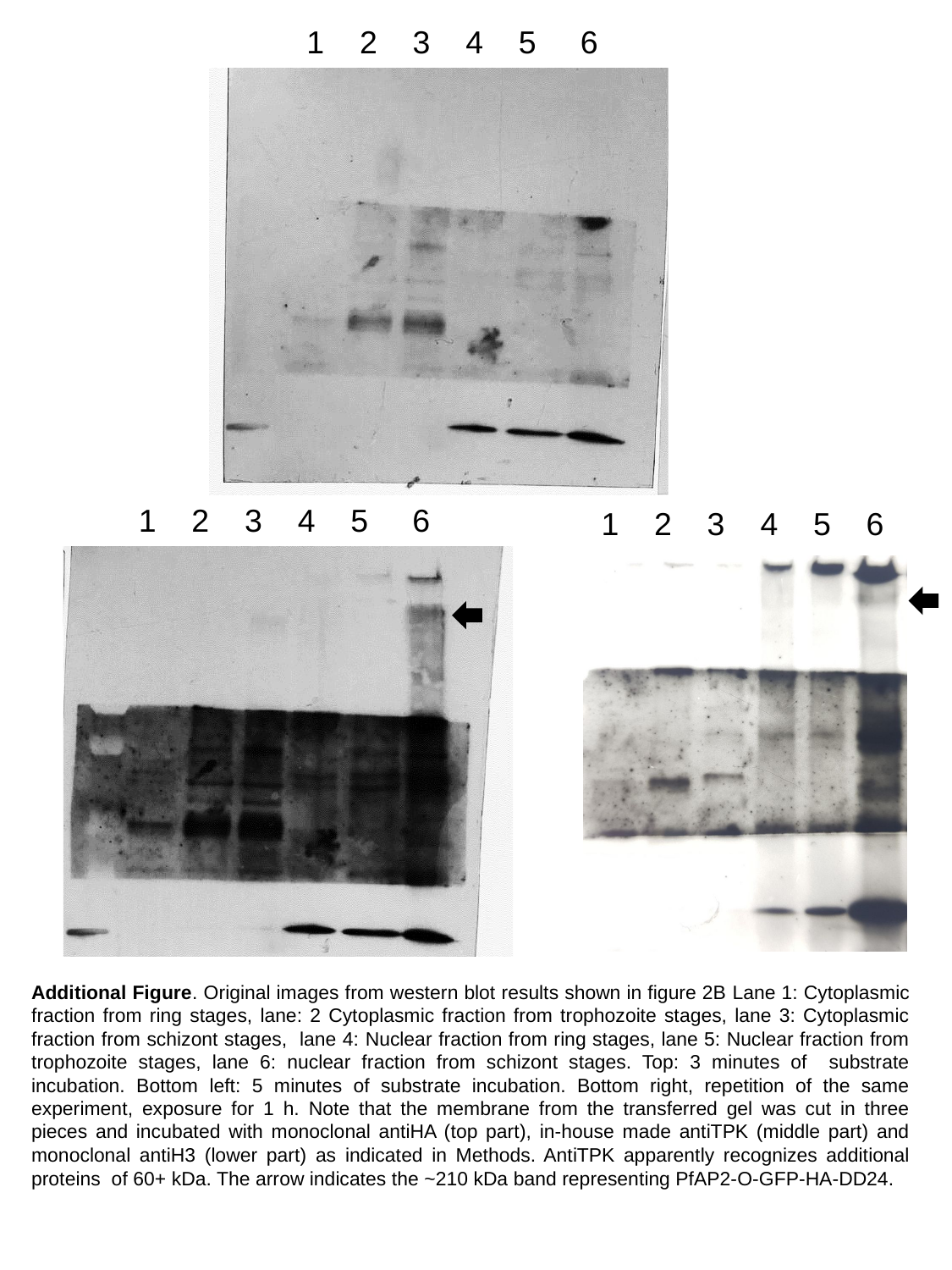

1 2 3 4 5 6
1 2 3 4 5 6
 1 2 3 4 5 6
Additional Figure. Original images from western blot results shown in figure 2B Lane 1: Cytoplasmic fraction from ring stages, lane: 2 Cytoplasmic fraction from trophozoite stages, lane 3: Cytoplasmic fraction from schizont stages, lane 4: Nuclear fraction from ring stages, lane 5: Nuclear fraction from trophozoite stages, lane 6: nuclear fraction from schizont stages. Top: 3 minutes of substrate incubation. Bottom left: 5 minutes of substrate incubation. Bottom right, repetition of the same experiment, exposure for 1 h. Note that the membrane from the transferred gel was cut in three pieces and incubated with monoclonal antiHA (top part), in-house made antiTPK (middle part) and monoclonal antiH3 (lower part) as indicated in Methods. AntiTPK apparently recognizes additional proteins of 60+ kDa. The arrow indicates the ~210 kDa band representing PfAP2-O-GFP-HA-DD24.
